# Diversified pattern of the human colorectal cancer microbiome

**DOI:** 10.1186/1757-4749-5-2

**Published:** 2013-03-07

**Authors:** Jiawei Geng, Hong Fan, Xiaodan Tang, Huiqin Zhai, Zhigang Zhang

**Affiliations:** 1 Department of Gastroenterology, The First People’s Hospital of Yunnan Province, Kunming, 650032, China; 2 State Key Laboratory of Genetic Resources and Evolution, Kunming Institute of Zoology, Chinese Academy of Sciences, Kunming 650223, China

**Keywords:** Chinese, CRC, Microbiome, Pyrosequencing

## Abstract

**Background:**

The aim of this study is to expand existing knowledge about the CRC-associated microbiome among Han Chinese, and to further discover the variation pattern of the human CRC microbiome across all population.

**Findings:**

Using pyrosequencing-based molecular monitoring of bacterial 16S rRNA gene from eight tumor/normal tissue pairs of eight Chinese CRC patients, we analyzed and characterized the basic features of the CRC-associated microbiome. Firstly, we discovered an increasing diversity among tumor-associated bacterial communities. Secondly, in 50% of Chinese CRC patients, we found a significant increase of *Roseburia* (*P* = 0.017), and a concurrent decrease of both *Microbacterium* (*P* = 0.009) and *Anoxybacillus* (*P* = 0.009) in tumor tissue.

**Conclusions:**

We discovered a novel CRC microbiome pattern in Chinese. Both the over-represented *Roseburia* bacteria at tumor sites and the over-represented *Microbacterium* and *Anoxybacillus* bacteria away from tumor sites were both closely related in Chinese CRC patients. Across several populations reported in this study and previously, we observed both common and distinctive patterns of human CRC microbiome’s association with a high-risk of CRC.

## Background

Bacterial infections play a potentially significant role in the pathogenesis of colorectal cancer (CRC) [1-5], though there are a variety of dietary, genetic, and environmental factors that add to CRC risk [6]. Previous studies however have reported that various infectious agents (e.g., *Fusobacterium spp*., *Bacteroides fragilis*, and *Escherichia coli*) are related to a high-risk of CRC across several different populations. These findings prompted us ask whether such associations between CRC and microbiome patterns are common or population-specific. Such a finding to this question is essential in developing personalized medicine strategies and treatment options for CRC patients.

The aim of this study was to quantitatively evaluate the differences of bacterial communities and compositions between eight tumor/normal pairs from eight Chinese CRC patients, as well as to characterize both the common and various patterns of the human CRC microbiome among different populations, ultimately to contribute towards a preliminary understanding of the bacterial driving forces at play in CRC.

## Methods

### Patients, sample collection, and DNA extraction

Eight Chinese CRC patients from Kunming, China were used in this study with (56.9±14.4) (SD) average age, (22.97±1.56) body mass index (BMI), and (1:1) male/female ratio. Four patients were diagnosed as having rectal cancers while the other half had colon cancers (ascending colon, transverse colon, descending colon, and sigmoid). From the eight patients, we obtained a total 16 tissue samples, including eight cancerous tissues and their matched adjacent normal tissues obtained via colonoscopy following the Standard Operating Procedures at the First People’s Hospital of Yunnan Province of China, Kunming. Participants were enrolled in the study prior to colonoscopy, and written informed consent was obtained from the patient for publication of this report and any accompanying images. Samples were removed endoscopically and immediately transferred from the colonoscopy room to the pathology suite and subsequently evaluated by the pathologist. Where possible one fragment of healthy tissue and one of tumor were chosen and placed in a cryotube, then frozen immediately in liquid nitrogen. All specimens were stored in their original tubes at -80°C prior to DNA extraction. This study conformed to the ethical guidelines outlined in the 1975 Declaration of Helsinki as reflected by a priori approval from the Medical Ethics Board of the First People's Hospital of Yunnan Province of China.

While frozen, an aliquot (~25 mg) of each specimen was suspended in a solution containing 200 ul buffer ATL (QIAGEN Kit Buffer for tissue lysis) and 200 ul of a slurry of 0.1-mm-diameter zirconia/silica beads (BioSpec Products, Bartlesville, OK). The mixed sample was then lysed by mechanical disruption with a bead beater (BioSpec Products), set on high for 2 min (20°C), followed by extraction with the QIAamp^®^ DNA Mini Kit (Qiagen, Inc., Valencia CA). DNA from tissue was eluted in a final volume of 200 ul elution buffer and stored at -20°C. Tubes containing only QIAamp^®^ DNA Mini Kit extraction controls were included throughout lysis and PCR to serve as negative controls.

### PCR amplification of V1-V2 hypervariable regions of 16S rRNA gene and pyrosequencing

The forward primer included the *454 Life Sciences* primer B sequence (5^′^ - CTATGCGCCTTGCCAGCCCGCTCAG -3^′^) and the broadly conserved bacterial primer 27 F (5^′^-AGAGTTTGATCCTGGCTCAG-3^′^). The reverse primer included the *454 Life Sciences* primer A sequence (5^′^-CGTATCGCCTCCCTCGCGCCATCAG- 3^′^), a unique 10-nt barcode used to tag each PCR product, and the broad-range bacterial primer 338R (5^′^- TGCTGCCTCCCGTAGGAGT-3^′^). Using the primer pair described above, triplicate PCR reactions were performed on each sample. Each resulting 25 ul reaction contained 0.2 μM forward and reverse primers, 3 μl template DNA, and 2.5 ul 10X PCR buffer plus Mg^2+^ (TaKaRa), 2.0 ul dNTP (2.5 mM each) (TaKaRa), 0.75 ul DMSO (100%), 0.25 ul TaKaRa Taq^™^ (5 U/μl). Thermal cycling was conducted at 95°C for 10 minutes, followed by 30 cycles of 95°C for 30 seconds, 52°C for 30 seconds, and 72°C for 90 seconds, with a final extension of 10 minutes at 72°C. Replicate amplicons were pooled and visualized on 1.5% agarose gels using EB stain in 0.5X TE. Amplicons were cleaned using MinElute^®^ Gel Extraction Kit (Qiagen) according to the manufacturer’s instructions. Amplicon DNA concentrations were determined using the Quant-iT PicoGreen dsDNA reagent and kit (Invitrogen). Using the amplicon pool, we carried out pyrosequencing using primer A and Titanium chemistry on a *454 Life Sciences* Genome Sequencer FLX instrument (Roche) at the DNA Sequencing Facility of the Kunming Institute of Zoology, Chinese Academy of Sciences.

### Bioinformatics analysis

Sequences were processed and analyzed using Qiime 1.4 [7]. Sequences were assigned to each sample by examining the 10-nt barcode based on the following criteria: a minimum (min) sequence length of 200 nt; maximum (max) sequence length of 400 nt; min qual score of 25; maximum number of errors in barcode of 0; maximum length of homopolymer run of 6; number of mismatches in primer of 0; excluding ambiguous and unassigned characters; and removed 454-adaptor B and 27 F bacterial primer from all assigned sequence data. Sequencing errors were removed from filtered sequences using denoiser 0.91 [8]. Using Chimera Slayer [9], chimera sequences arising from the PCR amplification were detected and excluded from the denoised sequences. The chimera-free sequences were then clustered into operational taxonomic units (OTUs) using CD-hit [10] with a criterion of a minimum identity of 97%. Representative sequences per OTU were classified using BLAST with default parameters in Qiime 1.4. All OTUs found in at least two samples were retained for performing the following further analyses.

### Statistical analysis

General characteristics were expressed as median and mean or percentages. Comparisons were performed between tumor and normal samples using the Mann-Whitney rank sum test or t-test in SigmaPlot 12.0 (Systat Software, Inc.). Statistical significance was set at *P* < 0.05.

## Results

**Figure 1:**
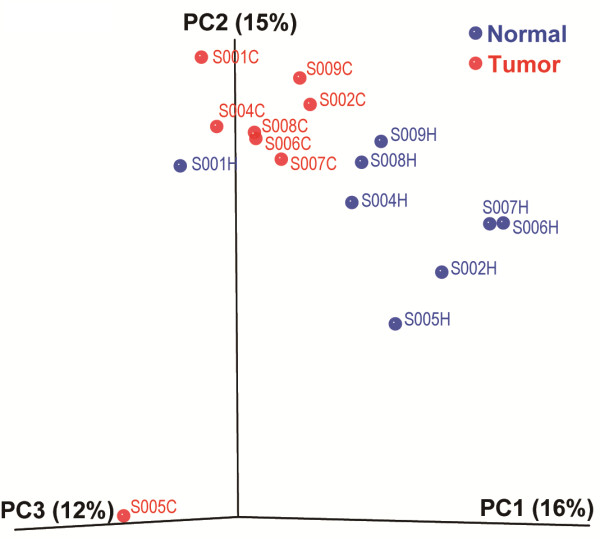
**16S rRNA gene surveys reveal hierarchical partitioning of human tumor tissue-associated microbiomes. **Bacterial communities were clustered using Principal Coordinate Analysis (*PCoA*) of the full-tree-based *Unifrac *matrix. Each point corresponds to a sample colored to indicate tumor or healthy status. Three principle components (PC1, PC2, and PC3) totally explained 43% of the variation. Sample name started with their corresponding studied patient number - S00X (X= 1, 2, 4, 5, 6, 7, 8, and 9), and the following tissue type (C stands for cancer tissue and H for matched adjacent health tissue).

We obtained a dataset consisting of 21,345 high-quality, classifiable 16S rRNA gene sequences with an average of 1334.1 ± 521.9 (SD) (n = 16) sequences per sample, after filtering raw data with our set of criteria (Methods). From the dataset, we identified a total of 410 OTUs, based on the conventional criterion of 97% sequence similarity (equal to species level), with an average of 138.9 ± 46.2 OTUs per sample (n = 16). Compared with normal results, the diversity index by both species richness (OTU number) and evenness (phylogeny-based Chao1) suggested an increasing trend of microbial diversity in tumors (mean; 122.3 ± 26.8 vs. 155.5 ± 56.8; 178.3 ± 41.0 vs. 230.7 ± 78.1). The significant difference of bacterial diversity was further confirmed by distinctive structural segregations of all 16 samples using PCoA analysis based on phylogeny-based *Unifrac* matrix (Figure 1).

**Figure 2:**
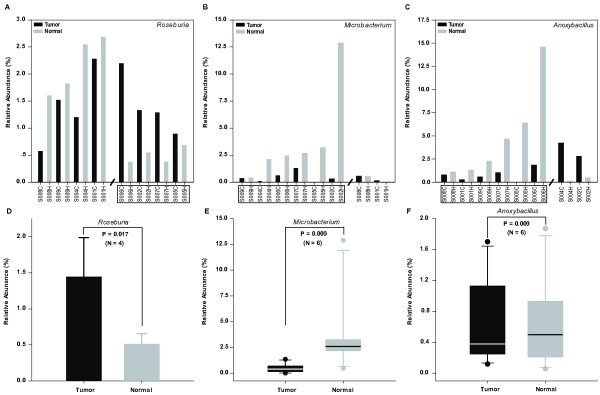
**Statistical comparisons of three dominant gut bacteria genera between tumor and normal tissues. A-C **respectively showed two different variation patterns of each of three dominant bacteria genera (*Roseburia*, *Microbacterium*, and *Anoxybacillus*)–either the overrepresentation in tumor sites or in normal sites (separated by slash). Those tumor/normal tissue pairs with significant differentiation of dominant bacteria abundance were marked by a box. **D**, significantly increasing *Roseburia *in tumor tissue (mean, t-test) corresponding to four tumor/normal pairs from 50% of patients marked by a box (**A**); **E**, significant decreasing *Microbacterium *in tumor (median, Mann-Whitney rank sum test) corresponding to six tumor/normal pairs from 75% of patients marked by box (**B**); **F**, significant decreasing *Anoxybacillus *in tumor (median, Mann-Whitney rank sum test) corresponding to six tumor/normal pairs from 75% of patients marked by a box (**C**). Sample names started with their corresponding studied patient number - S00X (X= 1, 2, 4, 5, 6, 7, 8, and 9) and the following tissue type (C stands for cancer tissue and H for matched adjacent health tissue).

By comparing the differences of bacteria components between eight tumor/normal tissues from eight Chinese CRC patients, we found two different variation patterns corresponding to each of three gut dominant bacteria genera (*Roseburia*, *Microbacterium*, and *Anoxybacillus*) (Figure 2A-2C), though each genus only showed one significant increasing or decreasing pattern in tumor tissue. Amongst 50% of patients, there was a significant increase (P = 0.017) of *Roseburia* in tumor samples (Figure 2A). Conversely, *Microbacterium* showed a significantly (P = 0.009) lower abundance in tumor than in normal tissue (Figure 2B) in 75% of patients. Similarly, in 75% of patients, *Anoxybacillus* also showed a significant decrease (P = 0.009) (Figure 2C) in tumors as compared with the level found in normal tissue. Similarly, there was a consistent underrepresentation of *Microbacterium* and *Anoxybacillus* in tumors observed in 50% of patients.

## Discussion

Our observations over the course of the study suggest that we have discovered a novel pattern to the human CRC microbiome among Han Chinese. According to the bacterial driver-passenger model for CRC put forward by Tjalsma *et al* (2012) [5], among 50% of Chinese CRC patients in this study, the over-represented *Roseburia* bacteria at tumor sites should in fact be considered as ‘passenger bacteria’ for CRC (previously known in other populations, but not previously observed in Chinese) and the over-represented *Microbacterium* and *Anoxybacillus* bacteria away from tumor sites—i.e., adjacent non-malignant tissue—as ‘driver bacteria’ for CRC (novel among all populations).

Consistent with several previous reports [3,4,11-13], we also found an increasing trend of *Fusobacterium spp.* in tumors among 87.5% of patients, though we did not discover a significant increase. Across several of the different, previously studied populations including Europeans, Americans, and Asians (in particular, Chinese and Vietnamese), a similar pattern was observed; such a consistent overrepresentation of *Fusobacterium spp.* in tumor tissue suggests that there exists a common mechanism of gut microbial disorder connected with CRC. This finding implies that it is crucial to begin defining an underlying association of the gut passenger bacteria *Fusobacterium* with an increasing risk of CRC for most populations, despite the lack of relative reports for many different populations to date.

We also found another gut bacterium, *Roseburia*, is potentially associated with an increased risk of CRC, due to the overrepresentation of *Roseburia* in tumor tissue among Chinese, as we noted in this study, and as previously reported among Dutch [13]. This finding is contrary to the study done by Wang *et al* study on another Chinese population [12] that compared the difference of stool flora between CRC patients and healthy subjects. Among the Chinese, the distinct discovery of *Roseburia* and its potential association with CRC may be due to different sample types, as another study done by Chen *et al* indicated a different microbial structure between the intestinal lumen and cancerous tissue in Chinese CRC patients. The microbial structure difference between the intestinal lumen and mucosa tissue in healthy subjects was further confirmed by Eckburg *et al*[14]. Additionally, two factors —diet and genetics—may have minor effects on the differentiation of *Roseburia* for CRC among Chinese, as we observed consistent overrepresentation of *Roseburia* in tumor tissues between the Chinese and Dutch, two populations with higher divergence of diet and genetics than within the Chinese population. Accordingly, the potential role of candidate gut passenger bacteria *Roseburia* should be emphasized more heavily during the occurrence of CRC, regardless of the population (e.g. Chinese or Dutch) and further investigated.

Similar to the relationship between CRC and gut bacteria *Fusobacterium* and *Roseburia* described above, the ‘passenger bacteria’ role of *Bacteroides* for CRC was supported by studies on the Chinese [12], Dutch [13], and French [15]. And the ‘driver bacteria’ role of *Bacteroides* was likewise confirmed by two other studies that examined a similar pattern among the Spanish, American and Vietnamese [3,4]. Furthermore, Wu *et al* confirmed that gut bacteria *Bacteroides fragilis* enables the promotion of colon tumorigenesis [1]. Similarly, based on *in vivo* experiments of mice model, *E. coli*-induced colitis is a driving factor of colorectal cancer [2], and the ‘passenger bacteria’ role of *Escherichia* species for CRC among the Chinese was also implied by the study done by Wang *et al*.. Moreover, besides the common pattern of the human CRC microbiome represented by gut passenger bacteria *Fusobacterium* within and between populations, there exists a diversified pattern in the human CRC microbiome due to three possible factors. One may be due largely to high variation of the normal human gut microbiome [16], potentially associated with diet [17,18], age [18,19], sample type (mucosa or stool) [14], host genetic factors [20], or other factors, such as antibiotic abuse [21]. Another factor may result from the different stage of tumor progression that is randomly selected by different studies, as the CRC microbiome variance may be temporally associated with developing tumors [5]. The last possible factor is that most findings to date have only been derived from bacterial 16S rRNA-based analyses, though some evidence from a metagenomic approach with a markedly more powerful ability to decipher the landscape of human CRC microbiome are intriguing [3,4]. Further studies of this kind will be helpful in confirming and elucidating the potential associations we have outlined in the present study.

In summary, in the present study we presented some initial findings the lead towards a deeper and more comprehensive view of the human CRC microbiome. The existing findings are suggestive of further research, and underscore the necessity of borrowing from both high-throughput meta-genomic or transcriptomic data and (animal) model experiments that will better define and validate the association of high-risk microbial populations with occurrence of CRCs across different populations.

## Abbreviations

CRC: Colorectal cancer; OTUs: Operational taxonomic units; rRNA: Ribosomal RNA.

## Competing interests

The authors declare that they have no competing interests.

## Authors’ contributions

JG performed research, analyzed data, and wrote the manuscript; HZ, XT, and HF performed research; ZZ conceived the study, performed research, analyzed data, and wrote the manuscript. All authors read and approved the final manuscript.

## Authors’ information

JG, HF, XT, HZ: Department of Gastroenterology, The First People’s Hospital of Yunnan Province, Kunming, China. ZZ: State Key Laboratory of Genetic Resources and Evolution, Kunming Institute of Zoology, Chinese Academy of Sciences, Kunming, China.

## Data sharing

The 454 sequence date set was deposited and available in the NCBI Sequence Read Archive (SRA) (accession number SRA060808).
